# Restructuring career counselling ventures of a mentoring program for medical students in the course of the COVID-19 pandemic

**DOI:** 10.3205/zma001366

**Published:** 2020-12-03

**Authors:** Jonathan A. Gernert, J. Zibold, L. J. U. Reik, T. Graupe, K. Dimitriadis

**Affiliations:** 1 University Hospital of Ludwig-Maximilians-University, Institute for Medical Education, Munich, Germany; 2 Ludwig-Maximilians-Universität (LMU), Klinikum der Universität München, Department of Neurology, Munich, Germany; 3 Ludwig-Maximilians-Universität (LMU), Klinikum der Universität München, Institute for Stroke and Dementia Research (ISD), Munich, Germany

**Keywords:** mentoring, mentors, medical education, online mentoring, COVID-19

## Abstract

**Objectives: **Due to the COVID-19 pandemic, medical curricula face major challenges. This also applies to mentoring programs, where face-to-face meetings are considered essential.

**Methods: **The LMU Munich medical faculty mentoring program (MeCuM-Mentor) adapted to counteract the unforeseen pause of conventional course formats and associated uncertainty of many students. We here present an approach to transform the established large scale or group mentoring events of our program into online formats. Three projects are presented as examples: 1. HowTo Klink (HK), mainly informative in nature and with peer-mentoring character, 2. FacharztDuell (FAD) and 3. “Auf ein Gespräch mit... (AEGM)”, both with a focus on career counseling.

**Results: **Initial evaluations show a similarly high participation rate and a high level of satisfaction among the participating students. Students' evaluation of whether the projects presented should take place in presence or in online format has so far shown no clear trend.

**Conclusion: **Prospective studies are necessary to investigate the effectiveness of these online formats and analyse differences in participant behaviour. The extent to which online mentoring can replace classic mentoring functions has to be discussed anew.

## Zusammenfassung

**Ziele: **Aufgrund der COVID-19-Pandemie stehen medizinische Curricula aktuell vor großen Herausforderungen. Dies gilt auch für Mentoring-Programme, bei denen persönlicher Austausch als wesentlich angesehen wird.

**Methoden: **Das Mentoring-Programm der Medizinischen Fakultät der LMU München (MeCuM-Mentor) wurde angepasst, um der unvorhergesehenen Pause der bisherigen Kursformate und der damit verbundenen Unsicherheit vieler Studierender entgegenzuwirken. Wir stellen hier einen Ansatz vor, um die etablierten Groß- oder Gruppen-Mentoring-Veranstaltungen unseres Programms in Online-Formate zu transformieren. Beispielhaft werden drei Projekte vorgestellt: 1. HowTo Klink (HK), wesentlich mit informativem sowie mit Peer-Mentoring-Charakter, 2. FacharztDuell (FAD) und 3. Auf ein Gespräch mit ... (AEGM), beide mit Schwerpunkt Berufsberatung.

**Ergebnisse: **Initiale Bewertungen zeigen eine ähnlich hohe Teilnahmequote und Zufriedenheit der Studierenden. Die Einschätzung der Studierenden, ob die genannten Projekte in Präsenz- oder Online-Format stattfinden sollen, hat bisher keinen klaren Trend gezeigt.

**Fazit: **Prospektive Studien sind erforderlich, um die Wirksamkeit dieser Online-Formate zu untersuchen und Unterschiede im Verhalten der Teilnehmer zu analysieren. Inwieweit Online-Mentoring klassische Mentoring-Funktionen ersetzen kann, muss neu diskutiert werden.

## Background

Over the past years, the importance of successful mentoring in health care and medical studies has been emphasized [1], [2]. According to Frei et al., personal interaction between mentor and mentee plays a crucial role in successful mentorship [3]. The mentoring program at the medical faculty of LMU Munich was designed and implemented in 2007 [4]. Since then, the program has developed two main pillars:

1. a one-to-one mentoring program with peer-mentors and medical professionals for more than 4.000 students [5],
2. a complementary second part, that comprises various projects and events with indirect mentoring character.

These projects can be categorized into informative events, panel discussions on career advice and group mentoring rounds. Although most of our projects were face-to-face events to encourage personal exchange between mentors and mentees, our program also used some online elements prior to the COVID-19 pandemic. These included an online newsletter with different extracurricular or curricular voluntary offers at the medical faculty, video podcasts with brief introduction of our mentors, an online matching tool and a website for the search of open medical doctoral dissertations [6]. In the course of COVID-19 pandemic, we were faced with the challenge to expand the online offer in a short time frame [7], [8]. 

## Project descriptions

In the following, we present three exemplary projects for different types of events:

1. *HowTo Klinik* (HK) is an informative event to introduce medical students to the clinical studies of our medical school and is held twice a year at the beginning of each semester (n=150). So far, HK took place in an auditorium, was informative in nature and enabled a subsequent informal exchange between students and peer-mentors to foster the mentoring character of the event.
2. *FacharztDuell *(FAD) is an event that highlights the differences between medical specialties in a moderated discussion with an additional question-and-answer session (ø 6 per year, n=100). The format is intended to advise medical students about their future academic careers [9].
3. *Auf ein Gespräch mit…* (AEGM) is a group-mentoring discussion (n=10) with selected guests representing various possible professional positions career paths after finishing medical school (ø 10 per year). Participants get inspired in regards of planning their own professional future and come into contact with medical mentors in casual atmosphere [10].

Due to the COVID-19 pandemic, all three projects were held for the first time as online video-events using the software Zoom Inc. hosted and supported by a team member of our mentoringprogram. HK was organized at the beginning of the semester. FAD was held once and focused on the role of psychiatrists, internists and virologists during the COVID-19 pandemic. In three AEGM events, students had the opportunity to discuss with physicians that followed a career in emergency medicine, from an internal and surgical perspective, as well as with doctors working as business consultants or in the pharmaceutical industry. Since our common advertising strategies (via announcements during lectures or posters on campus) were not possible, all events were advertised only online via social media channels and on our website. LMU medical students from all semesters were able to apply for all three projects in parallel. For data protection reasons and in contrary to face-to-face events held in an auditorium, registration with university email address on our website was required for all three formats. We were therefore able to ensure that only authorized persons participated. Students were able to submit questions in advance via our website. In addition, participants could ask question during the event via chat or personally (in events with fewer students, like AEGM). The moderators categorized questions and tried to ask representative ones from different themes. FAD and AEGM were evaluated online. 

## Results

The above-mentioned events were attended by 120 (HK), 105 (FAD) and in average eight (AEGM). The duration of both, face-to-face and online events exceeded the planed 1,5 hour and were therefore terminated by the moderator after about 2h. The rate of responses for the evaluations was 29% (FAD), 84% (AEGM). The overall event was rated (Likert-scale: 1=very good, 6=insufficient) with 1.08±0.28 (FAD) and 1.19±0.40 (AEGM), the organization with 1.33±0.48 (FAD) and 1.52±0.81 (AEGM) respectively. The number of participants was rated appraised as appropriate by all students in both formats. When asked whether online or face-to-face events is the most suitable format for the event, 56% of the FAD participants voted for face-to-face meetings, whereas 57% of AEGM participants preferred an online format.

## Discussion

We demonstrated that different mentoring projects can be converted into an online format. Number of participants in online events are comparable to face-to-face events. Evaluation results showed a high level of satisfaction among students. A number of mentoring programs offer personal mentoring as well as virtual mentoring formats in parallel [11], [12]. However, some criticise that exclusive e-mentoring does not meet the definition of mentoring due to the lack of a personal encounter [3]. The effectiveness of online events versus face-to-face has to be evaluated in prospective studies. 

## Conclusion

First preliminary results of our events lead us to conclude that online formats can represent an adequate alternative to face-to-face ones. Nevertheless, long-term evaluations are still pending. These aim to answer the question of whether our online formats meet characteristics of mentoring and what effects video-based events have on the depth of conversation. Advantages and disadvantages of e-mentoring events compared to face-to-face mentoring events must therefore be analysed in order to better estimate the appropriate mix in the portfolio mentoring programs offer. 

## Competing interests

The authors declare that they have no competing interests. 
